# The Age at Which Insanity Is Most Prevalent

**Published:** 1846-08

**Authors:** 


*The Age at which Insanity is most Prevalent.*—To determine the period of life which furnishes the greatest number of insane persons, it is sufficient to bring together the records, made up under different circumstances. One of them, made at the Bicètre, where poor men are received; another, at the Salpêtrière, a hospital destined for poor women; the third, at an establishment devoted to the wealthy. From these reports we may conclude:—1st, that the age which furnishes the greatest number of insane, is, for men, that from 30 to 40 years; whilst for women, it is that from 50 to 60 years; 2nd, that the ages which furnish the least, are, for both sexes, childhood, youth, and advanced age; 3rd, that among women, insanity appears earlier than among men, indeed from 29 to 30 years of age; 4th, that the rich are afflicted, in comparison with the total number of insane persons, in a greater proportion than the poor.—*London Lancet.*

